# Supplementary material for: Learning new perceptual skills: Individual differences in the computations that integrate novel sensory cues into depth perception
Source: iScience. 2026 Mar 27;29(5):115526. doi: 10.1016/j.isci.2026.115526 (PMC13091759; doi:10.1016/j.isci.2026.115526)
Supplement: Document S1. Figures S1 and S2 [file mmc1.pdf]

iScience, Volume *29*

Supplemental information

Learning new perceptual skills: Individual differences in the computations that integrate novel sensory cues into depth perception

Meike Scheller, Stacey Aston, Thomas Chazelle, Chris Allen, Heather Slater, and Marko Nardini

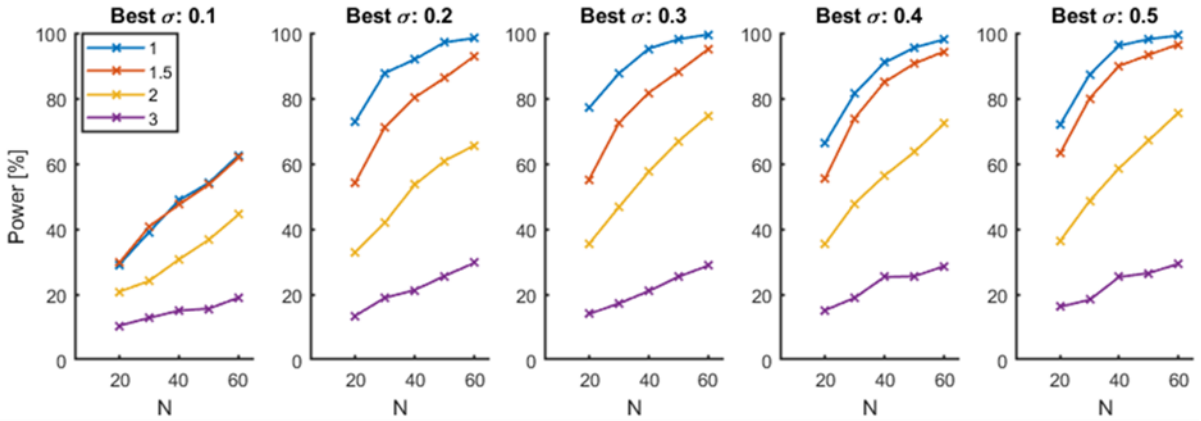

Supplemental figure 1. Power estimates
Power, defined as the probability of finding a true cue combination effect, as a function of sample size, the best sensory noise level, and sensory noise ratios between the cues.

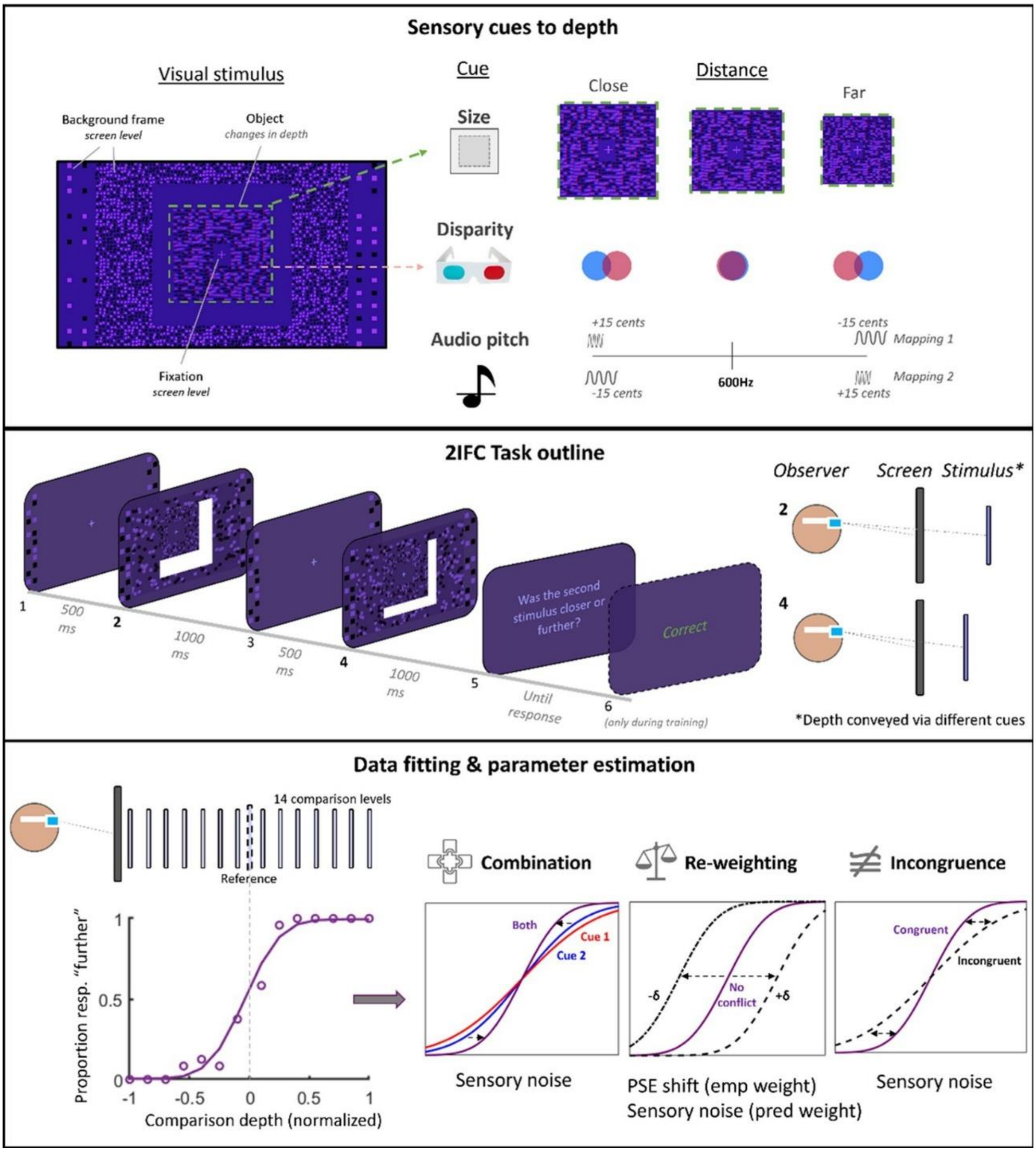

Supplemental figure 2. Experimental methods and design.
Top: Stimuli comprised visual (size, disparity; familiar) and auditory (pitch; novel) cues to depth. The familiar cues followed a natural mapping of depth, based on the physics of real-world cue behaviour. With increasing distance, the object would become smaller (size cue). The offset between left and right image would increase with increasing distance from the screen, with the directionality of the offset indicating the stimulus being closer or further than the screen (disparity cue). The novel audio cue was mapped to depth with either increasing pitch or decreasing pitch with further distance. The mapping direction was counterbalanced across participants. *Middle*: Temporal outline of the 2IFC task. Two stimuli were presented after each other at different levels of depth, indicated in the sketch on the right, with a 500ms ISI. Depth was conveyed via individual cues or via two cues at the same time. *Bottom*: Sensory noise parameters were estimated by fitting psychometric functions (PF) to the proportion of trials on which participants responded that a specific comparison stimulus (different levels represented in pale purple in the sketch above) was further than the reference stimulus (presented with a dashed outline in the sketch above, aligned with 0 of the PF). To assess combination, we estimated the sensory noise of the single and combined cue conditions from

changes in the PF slope (equation 5 in main text). To assess re-weighting, we estimated the absolute mean shift in PSEs of the conflict condition PFs, relative to the no conflict condition. Weights for the disparity cue were derived from these shifts relative to the offset (δ; equation 7 in main text). Also, sensory noise was estimated for the single cue conditions to assess the relative predicted weight for the disparity cue (equation 8). To assess incongruence sensitivity, we estimated the sensory noise of the combined congruent and combined incongruent conditions.
